# Management of Kidney Stone Disease in Pregnancy: A Practical and Evidence-Based Approach

**DOI:** 10.1007/s11934-022-01112-x

**Published:** 2022-10-05

**Authors:** Patrick Juliebø-Jones, Bhaskar K. Somani, Stephen Baug, Christian Beisland, Øyvind Ulvik

**Affiliations:** 1 Department of Urology, Haukeland University Hospital, Bergen, Norway; 2 Department of Clinical Medicine, University of Bergen, Bergen, Norway; 3 Department of Urology, University Hospital Southampton, Southampton, UK

**Keywords:** Pregnancy, Urolithiasis, Renal colic, Ureteroscopy

## Abstract

**Purpose of Review:**

Suspected kidney stone disease during pregnancy is a difficult condition for health professionals to manage. This is partly due to the more limited range of diagnostic and therapeutic strategies, which can be safely applied. A comprehensive review of literature was performed to identify evidence to develop a practical guide to aid clinicians.

**Recent Findings:**

Ultrasound remains the recommended first line option for imaging. Complicated cases, such as suspected infected obstructed system, require urgent decompression such as in the form of percutaneous nephrostomy. This article highlights the pharmacotherapeutic agents, which are considered safe for use in pregnancy. Where surgical intervention is indicated, evidence supports ureteroscopy to be a safe option as long as infection has been treated. Ureteroscopy can offer definitive clearance of the stone(s) and can be less burdensome regarding bothersome symptoms compared to indwelling ureteral stent or nephrostomy, which also require regular exchange due to the high propensity for encrustation in pregnancy.

**Summary:**

A multidisciplinary approach is fundamental to safely manage suspected kidney stone disease in pregnancy. Adoption of a locally agreed pathway as suggested in this article supports improved patient care.

## Introduction

Kidney stone disease (KSD) complicates 1:200 to 1:2000 pregnancies and is the commonest non-obstetric cause for acute hospital admission(s) [1]. In such cases, the urologist is treating for two, and the clinical challenge is heightened accordingly [2]. The importance of timely and precise management cannot be overstated given the risk of adverse and even fatal sequelae, which include preterm labour and miscarriage among others [3]. Initial misdiagnosis occurs in up to one-third of cases and subsequent treatment delays are not uncommon [4].

Often presenting acutely and out of hours, this clinical scenario can be difficult to navigate as a result of a multitude of factors. This includes restrictions in diagnostic imaging and a limited profile of safe pharmacotherapies. Furthermore, evidence used to direct patient care is nearly all derived from lower levels than are optimal. Despite these limitations, the continued advancement of surgical technology and expertise has delivered a number of subtle changes to the management pathway, which allows for improved patient care accordingly [5]. While international guidelines do make reference to KSD in pregnancy, often it is abridged and limited to key recommendations only [6]. Therefore, it can be a challenge for the time pressured clinician to find a go-to resource for use on a day-to-day basis.

Our aim was to deliver such a practical framework to guide the clinician based on recent evidence from world literature, which is supplemented with experience and lessons learned from two tertiary endourology centres.

## Methods and Materials

A comprehensive search of literature was performed. Bibliographic databases searched included Medline, Google Scholar and Scopus. Search terms included but were not limited to ‘pregnancy’, ‘renal colic’ and ‘urolithiasis’. All article types were considered. International guidelines were also consulted. The gathered evidence was reviewed by the authors and led to the development of a practical and multidisciplinary guide for clinicians.

### Epidemiology

In 2020, a study of 1.4 million women by Sohlberg et al. revealed KSD to be diagnosed in 1% of all pregnancies [7]. Furthermore, multivariate analysis revealed a significantly higher risk of pyelonephritis in these subjects as well as an increased risk of spontaneous abortion and foetal prematurity. A recent 10-year retrospective study revealed risk of gestational diabetes and pre-eclampsia to be significantly more common among stone formers (*p* < 0.002) [8]. Furthermore, this was independent of pre-existing diabetes, hypertension and chronic kidney disease (CKD). Findings from the National Health and Nutrition Examination Survey (NHNES) highlight that a prior pregnancy doubles the subsequent risk of KSD [9]. KSD during pregnancy is also associated with significantly higher recurrence rates post pregnancy compared to those with no history of KSD (12.5% vs. 0.4%, *p* < 0.0001) [10•].

### Anatomical and Physiological Changes During Pregnancy

As a result of the enlarging uterus, gestational hydronephrosis occurs by the third trimester in up to 90% and 67% in the right and left side, respectively [11]. Indeed, it can occur as early as the 6th week of pregnancy and persist until 6 weeks after delivery [12]. Even when not caused by KSD, hydronephrosis in pregnancy can be painful for the patient and therefore may itself be a cause for the patient’s acute presentation. Dilatation is not usually observed below the pelvic brim and therefore an obstruction below this level raises the suspicion of intraluminal obstruction secondary to KSD. This dilatation also serves to increase the risk of stone migration and subsequent obstruction [13]. Stones are twice as likely to be located in the ureter than kidney when diagnosed during pregnancy [5]. Prolonged dilation results in urinary stasis, which, together with elevated progesterone levels, reduces ureteral peristalsis and promotes formation of urinary crystals. This is further accelerated by the gestational increase in glomerular filtration rate (GFR) and plasma flow (up to 50%), which leads to increased excretion of uric acid, oxalate and sodium [14]. Other lithogenic factors related to pregnancy include elevated urine pH and hypercalciuria. The latter occurs as a result of increased GFR and placental production of 1,25-dihydroxycholecalciferol to meet requirements of the foetus [15]. However, excretion of inhibitors to stone formation such as glycoprotein, nephrocalcin and urate is believed to compensate for the aforementioned lithogenic properties [3]. Calcium phosphate is the commonest stone composition type in contrast to calcium oxalate in general population [13].

### Presentation and Diagnosis

A recent systematic review by Dai et al. concluded the commonest presenting symptoms of KSD in pregnancy were flank pain (80–100%), nausea/vomiting (20–69%), haematuria (non-visible: 57–94% and visible: 15–23%) and fever/chills (7–11%) [2]. In addition to this, their results confirmed that diagnosis usually occurs during the second (38%) and third trimester (48%). Patients may also present to hospital due to an obstetric complication of the stone event such as pre-eclampsia [16].

While a number of nomograms exist in urology to predict likelihood of a patient attending the emergency department with a ureteral stone, e.g. STONE, none has currently been validated for use in pregnancy [17, 18•].

### Investigation

Patients should undergo a clinical history and physical examination. Urinalysis should be performed and sent for culture testing as required. Standard blood tests should be performed with initiation of a sepsis protocol as required. It is imperative that all pregnant patients referred also undergo an urgent gynaecological assessment in order to confirm the healthy status of the foetus as well as rule out an obstetric cause for the pain and/or an obstetric complication of a stone event. N’gamba et al. reported that among 82 pregnant patients referred acutely with suspected renal colic, only 29.3% were found to have a stone after further investigation [19]. This highlights the important role that additional tests can serve [15].

### Imaging

#### Ultrasound (US)

Foetal exposure to radiation is harmful and can render stochastic (carcinogenesis) and non-stochastic (teratogenesis) sequelae [20]. It should therefore be avoided unless absolutely necessary. International guidelines recommend US as first-line investigation accordingly [6]. Attention should be paid to determine the following: hydronephrosis, dilatation of the distal ureter and ureteric jets. Absence of the latter carries sensitivity and specificity of 100% and 91%, respectively, for diagnosing unilateral obstruction due to KSD [21]. However, ureteric jets can be missing in 15% of pregnant women so interpretation in light of other diagnostic findings is recommended [22]. Doppler US can be used to measure the renal resistive index (RI) (peak systolic velocity – end diastolic velocity] / peak systolic velocity), which helps to further distinguish possible causes of the dilated urinary system [23]. Gestational hydronephrosis does not result in an elevated RI; however, obstruction caused by ureteric obstruction does (sensitivity 45%, specificity 91%) [24, 25]. Transvaginal ultrasound can be useful to supplement abdominal US, especially if the latter is inconclusive, and can help identify a distal ureteric stone [26]. However, in addition to body habitus, operator dependency is a limiting factor associated with US and the sensitivity for KSD in pregnancy ranges from 34 to 92.5% [27].

When requesting the ultrasound, it is important to highlight and detail the additional information which may not be routinely performed among general population, e.g. presence of ureteric jets. It is also important to relay the need for the patient to attend such a scan with a full bladder as this can be overlooked and result in less than satisfactory views at US.

#### Magnetic Resonance Imaging (MRI)

MRI using T2-weighted images (without intravenous contrast) does serve as an option to help differentiate physiological and pathological hydronephrosis in pregnancy. While there do exist theoretical risks associated with MRI such as thermal effect of radiofrequency pulses, the American College of Radiology have determined it to be safe (1.5 T) in all pregnant patients and it carries a sensitivity and specificity for diagnosing obstructive stone disease of 77% and 83%, respectively [27, 28]. MRI in this setting does hold disadvantages including more limited availability, especially out of hours as well as no clear stone signal and prolonged acquisition time. MRI cannot visualise a stone, rather it may appear as a signal defect below a standing column of high signal urine sitting in a dilated ureter [29]. The calibre of the ureter may cut off suddenly in its lower portion rather than taper as it comes towards the bladder. This may be accompanied by peri-renal oedema and high-intensity fluid [30]. MRI can also serve to visualise other causes for the abdominal pain such as appendicitis. Several protocols have been described such as thin-slice, fast spin echo (FSE) for detection of small stones [31]. An alternative is the half-life Fourier singe-shot turbo spin-echo (HASTE) protocol, which can be completed in less than 15 min [32].

### Computed Tomography (CT)

The role of CT, e.g. ultra-low dose (<1.9 mSv) in pregnancy, has been studied in world literature [29]. While this imaging modality has been shown to yield a high positive predictive value (95%), the true risks to the foetus remain largely unknown and conclusions drawn are largely hypothetical. International guidelines do recognise CT as a last line imaging option for this special population [6]. However, given there are still issues regarding the potential for harmful sequelae, we do not include it at all in our diagnostic pathway. The rationale for this is to eliminate any unwanted risk to the foetus [30]. If a CT is to be performed, the most important time period to avoid is the 2nd to 15th week of gestation when radiation effects on the foetus are highest. Most centres do not have a set CT protocol for this clinical scenario. This is largely due to its rarity but also, such a protocol needs to be adapted to specifics such as mother’s weight, which changes over the course of the pregnancy. Patient counselling and involvement in the decision-making process surrounding CT should also occur.

## Treatment

### Conservative

Conservative management is adopted in the first instance unless the patient’s condition mandates emergency decompression via insertion of percutaneous nephrostomy tube or cystoscopy and placement of ureteral stent. Expectant management with re-hydration, analgesia, anti-emetics and close observation results in successful spontaneous stone passage in 23–84% of cases [2].

#### Analgesia

While paracetamol is safe in pregnancy, NSAIDs are contraindicated due to risk of premature closure of the ductus arteriosus as well as premature oligohydramnios and spontaneous abortion [20]. Low-dose and short-term morphine, e.g. morphine sulphate, is considered safe for pregnant females; however, when given in higher doses and over a long duration, it can be associated with foetal narcotic addiction, retardation of intra-uterine growth and premature labour.

#### Anti-emetic

Guidance from the Royal College of Obstetricians and Gynaecologists (RCOG) outlines several agents, which have no documented adverse effects to the foetus [33]. These include antihistamines such as cyclizine and promethazine, phenothiazines such as prochlorperazine and dopamine antagonists such as domperidone and metoclopramide. However, the latter can be associated with extra-pyramidal side effects and so are recommended as a second-line agent. This also applies to ondansetron because there is more limited data on its use in pregnancy. There are validated tools for assessment of nausea and vomiting in pregnant women such as the Pregnancy-Unique Quantification of Emesis (PUQE) index [34].

#### Antibiotics

Where antibiotics are indicated, penicillin and cephalosporins are the safest choices in contrast to erythromycin (maternal cholestasis), sulfonamides (neural tube defects), nitrofurantoin (foetal anaemia), tetracycline (bone defects), chloramphenicol (circulatory collapse—‘grey baby syndrome’), aminoglycosides (foetal and CNS toxicity) and quinolones (bone defects) [20].

#### Alpha Blockers

While previous meta-analyses have concluded that there may be a role for alpha-blockers as medical expulsive therapy (MET) for distal ureteric stones > 5 mm, debate regarding its use in the real-world setting continues and consensus is lacking [35]. A recent retrospective study of pregnant patients who received MET revealed no significant increase (*p* = 0.18) in stone passage rate compared to the control group and also no reduction in the need for surgical intervention. Based on such findings and the added risk of adverse events, MET is not routinely used for clinical practice for KSD in pregnancy [36].

### Surgical Intervention

Approximately 30% of pregnant patients with KSD will require intervention of some kind [20]. The ultimate choice of intervention modality should be tailored to the individual patient as well as the local expertise.

#### Emergency Decompression

Patients with acute renal failure and/or signs of sepsis should undergo immediate decompression via insertion of percutaneous nephrostomy (PCN) or cystoscopy and placement of ureteral stent. Ultrasound can be used rather than fluoroscopy to confirm placement. Definitive treatment of the stone should follow at a later date.

#### Temporising Measures

Traditionally, PCN or ureteral stent insertion has also been adopted to manage all patients with symptomatic KSD diagnosed during pregnancy where conservative measures have failed. However, it is problematic as the greater rates of encrustation in pregnancy often require stent exchange every 4 to 6 weeks [37]. PCNs and indwelling ureteral stents can also have a deleterious effect on quality of life. In a 15-year retrospective review of all pregnant patients with KSD at their institution, Rivera et al. found that 47% of patients with ureteral stent required early induction due to stent intolerance [38]. This reinforces the merits of definitive stone treatment where conservative measures have failed. However, it is appreciated that depending on the setting and local expertise, this may not be possible and referral to specialist centre may be necessary.

#### Shockwave Lithotripsy (SWL)

While there are studies revealing cases of SWL having been delivered inadvertently during pregnancy and to no ill effect to the foetus, it is contraindicated and not part of current clinical practice due to potential risk to the foetus [39].

#### Ureteroscopy

First described in setting of pregnancy over 20 years ago, URS now represents the surgical intervention of choice to achieve definitive stone clearance in pregnant patients [40]. The majority of centres perform URS using general or spinal anaesthesia; however, local anaesthesia ± sedation has been reported as a safe alternative in appropriately selected cases [1]. It is especially important to avoid general anaesthesia in the first trimester as use of volatile gases carries risk of causing morphogenetic anomaly [41].

Systematic review by Ishii et al. evaluated outcomes from 271 procedures over a 22-year period and revealed an overall stone-free rate of 85% [1]. However, the complication rate was 16.1%, which underlines the need to maximise attention to detail and tailored surgical care. With the introduction of newer generation laser systems such as thulium fiber laser (TFL), which holds advantages such as reduced operative time, the role of URS in pregnancy may be expanded even further [42, 43••].

### Percutaneous Nephrolithotomy (PCNL)

While there have been several case reports (less than 20 in world literature to date) of successful PCNL being performed in pregnancy, it is not currently part of standard practice not least because of the difficult patient positioning, e.g. prone, need for general anaesthesia and fluoroscopy [41].

### Additional Considerations

#### Role of a Clear Local Care Pathway

**Fig. 1:**
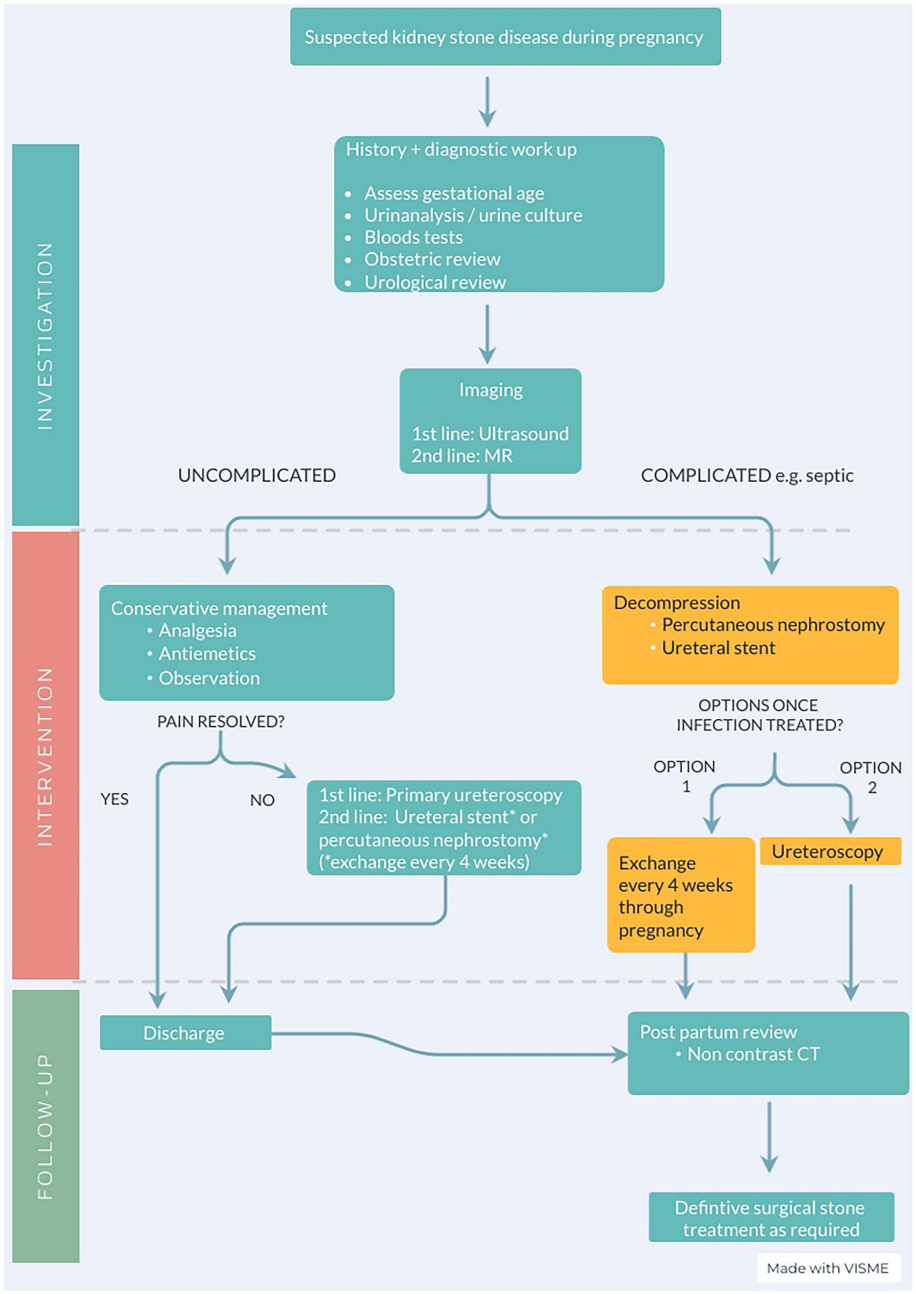
Management pathway for suspected kidney stone disease in pregnancy

Unwanted delays in the community or emergency department can occur in cases where there is a lack of direction regarding the specialty under which the patient should be admitted, e.g. obstetric or urology. Therefore, clear local guidelines and protocols are recommended to help avoid this (Fig. 1).

## Conclusion

Managing the pregnant patient with KSD is challenging. It demands clear communication and close collaboration between urologist and obstetric team. An understanding of the condition and adopting a stepwise approach can lead to a successful resolution of the clinical problem. Local implementation of a management pathway such as that outlined in this article can help navigate this complex clinical scenario and deliver a safe outcome for both mother and unborn child.
